# Activity and Cell-Death Pathway in *Leishmania infantum* Induced by Sugiol: Vectorization Using Yeast Cell Wall Particles Obtained From *Saccharomyces cerevisiae*

**DOI:** 10.3389/fcimb.2019.00208

**Published:** 2019-06-14

**Authors:** Débora Botura Scariot, Hélito Volpato, Nilma de Souza Fernandes, Edna Filipa Pais Soares, Tânia Ueda-Nakamura, Benedito Prado Dias-Filho, Zia Ud Din, Edson Rodrigues-Filho, Adley Forti Rubira, Olga Borges, Maria Do Céu Sousa, Celso Vataru Nakamura

**Affiliations:** ^1^Laboratory of Technological Innovation in Drugs and Cosmetics Development, State University of Maringá, Maringá, Brazil; ^2^Faculty of Pharmacy, University of Coimbra, Coimbra, Portugal; ^3^Chemistry Department, Federal University of São Carlos, São Carlos, Brazil; ^4^Chemistry Department, State University of Maringá, Maringá, Brazil; ^5^CNC - Center for Neurosciences and Cell Biology, University of Coimbra, Coimbra, Portugal

**Keywords:** yeast cell wall particles, sugiol, glucan, macrophage, antileishmanial activity

## Abstract

Visceral leishmaniasis, caused by *Leishmania infantum*, is a neglected tropical disease, to which efforts in the innovation of effective and affordable treatments remain limited, despite the rising incidence in several regions of the world. In this work, the antileishmanial effects of sugiol were investigated *in vitro*. This compound was isolated from the bark of *Cupressus lusitanica* and showed promising activity against *L. infantum*. In spite of the positive results, it is known that the compound is a poorly water-soluble diterpene molecule, which hinders further investigation, especially in preclinical animal studies. Thus, in an alternative delivery method, sugiol was entrapped in glucan-rich particles obtained from *Saccharomyces cerevisiae* yeast cell walls (YCWPs). To evaluate the activity of sugiol, the experiments were divided into two parts: (i) the *in vitro* investigation of antileishmanial activity of free sugiol against *L. infantum* promastigotes after 24, 48, and 72 h of treatment and (ii) the evaluation of antileishmanial activity of sugiol entrapped in glucan-rich particles against intracellular *L. infantum* amastigotes. Free sugiol induced the cell-death process in promastigotes, which was triggered by enhancing cytosolic calcium level and promoting the autophagy up to the first 24 h. Over time, the presence of autophagic vacuoles became rarer, especially after treatment with lower concentrations of sugiol, but other cellular events intensified, like ROS production, cell shrinkage, and phosphatidylserine exposure. Hyperpolarization of mitochondrial membrane potential was found at 72 h, induced by the mitochondria calcium uptake, causing an increase in ROS production and lipid peroxidation as a consequence. These events resulted in the cell death of promastigotes by secondary necrosis. Sugiol entrapped in glucan-rich particles was specifically recognized by dectin-1 receptor on the plasma membrane of macrophages, the main host cell of *Leishmania* spp. Electron micrographs revealed particles containing sugiol within the infected macrophages and these particles were active against the intracellular *L. infantum* amastigotes without affecting the host cell. Therefore, the YCWPs act like a Trojan horse to successfully deliver sugiol into the macrophage, presenting an interesting strategy to deliver water-insoluble drugs to parasitized cells.

## Introduction

Neglected tropical diseases (NTDs) remain a global health policy challenge due to the large proportion of the world's population at risk, and the lack of therapeutic development and dedicated drug innovation. More than one billion people are affected by NTDs, the majority living in tropical or subtropical areas, and in war zones, such as Syria (Al-Salem et al., 2016). Even in the face of this alarming scenario, only 4% of the new drugs and vaccines that were registered between 2000 and 2011 were for use in the treatment or prevention of NTDs (Pedrique et al., 2016). NTDs affect the poorest populations, therefore pharmaceutical industries have not shown a strong commitment to the development of novel drugs to combat NTDs due to a lack of financial return (Trouiller et al., 2002; Field et al., 2017). Among these diseases recognized by the World Health Organization as NTDs, three of them belong to the Trypanosomatidae family of parasites: Chagas' disease, African trypanosomiasis, and leishmaniasis.

The conventional therapies to treat leishmaniasis available are the pentavalent antimonials, amphotericin B, and Miltefosine. The serious side effects caused by these therapies result in the early abandonment of treatment, which has led to the appearance of resistant parasite strains. Thus, novel effective molecules and strategies for treating leishmaniasis are necessary to provide a safer treatment and better quality of life for patients, and also to prevent the emergence of resistant strains. The goal of finding new molecules that act against *Leishmania* species is hindered by the poor aqueous solubility of the majority of natural or synthetic compounds, which jeopardizes their application due to issues in the pharmacokinetic aspects (Burton et al., 2002; Pouton, 2006; Vasconcelos et al., 2007). It is estimated that about 40% of the tested bioactive substances present problems related to low water solubility (Savjani et al., 2012), including natural products (Coimbra et al., 2011). This is the case of sugiol, a diterpenoid isolated from the bark of several trees from the Cupressaceae family, such as *Cupressus sempervirens* (Zhang et al., 2012)*, Cupressus geovania* (Jolad et al., 2005)*, Cupressus lusitanica* (Mohareb et al., 2010)*, Calocedrus formosana* (Chao et al., 2005)*, Metasequoia glyptostroboides* (Bajpai and Kang, 2014), and *Pilgerodendron uviferum* (Solís et al., 2004). Despite unsatisfactory water solubility previously reported (Sengupta et al., 1960), sugiol has already been shown to have anti-inflammatory, antibacterial, and anti-cancer properties during *in vitro* assays (Bajpai and Kang, 2011; Sengupta et al., 2011; Jung et al., 2015; D'yakonov et al., 2017).

Nowadays, the efforts in the drug discovery field are focused on directing drugs to the target cell using drug carriers, in order to reduce the total daily dose required to treat diseases (Date et al., 2007; Paramera et al., 2014; Shaw and Carter, 2014). To achieve this, the compound may be incorporated in liposomes, microspheres, gels, cyclodextrins, and polymer based-particles, like nano and microparticulate systems (Tiwari et al., 2012). The high cost of encapsulated therapies is the major obstacle in this market and could compromise the success of future innovations in this field (Bosetti, 2015). The amphotericin B liposome is the state-of-the-art strategy to treat leishmaniasis, being more effective and presenting low toxicity compared to the standard amphotericin B treatment. However, this treatment can be up to 50 times more expensive than the conventional therapy (Rex and Walsh, 1999; Assis et al., 2017; de Carvalho et al., 2017).

In this study, baker's yeast or *Saccharomyces cerevisiae*, an affordable raw material, was used to obtain glucan particles derived from the yeast cell wall, after the removal of the intracellular material, proteins, and chitin (Soto and Ostroff, 2008). The yeast cell wall particles (YCWPs) derived from this process were used to carry sugiol into the *Leishmania-*infected macrophages. The rich β-1,3-D-glucan composition of the yeast cell wall ensures its phagocytosis after recognition by the dectin-1 receptor on the plasma membrane of fibroblasts, dendritic cells, and macrophages. Signaling cascades are activated promoting a set of responses inside the cell, including phagocytosis, oxidative burst, and the production of inflammatory mediators, cytokines, and chemokines that activate other immune cells (Soto and Ostroff, 2008; Goodridge et al., 2009; De Smet et al., 2014). YCWPs may be digested within phagosomes, similar to what happens with viable yeasts, releasing the entrapped drug within the cell (Saijo et al., 2007; Taylor et al., 2007; Lord and Vyas, 2019). This is an interesting strategy to deliver insoluble compounds directly to the target cells, overcoming the obstacle related to solubility and permeability (Burton et al., 2002; Yan et al., 2005; Figueiredo et al., 2011; Vetvicka, 2011).

YCWPs have been reported as a DNA delivery system and as carriers for sensitive magnetic resonance imaging (MRI) probes (Soto and Ostroff, 2008; Figueiredo et al., 2011; Soares et al., 2018). Moreover, phase I and II clinical trials using loaded YCWPs as a vaccine against metastatic melanoma, due to their reported immunomodulatory properties, are currently in progress (https://clinicaltrials.gov/ct2/show/NCT02678741;https://clinicaltrials.gov/ct2/show/NCT02301611).

In this study, we have evaluated the activity of sugiol against *L. infantum*, one of the causative agents of visceral leishmaniasis or kala-azar. Antileishmanial activity was assayed *in vitro*, and morphological and ultrastructural alterations were evaluated. The effect of free sugiol on cellular pathways, through the formation of autophagic vacuoles, generation of reactive oxygen species (ROS), lipid peroxidation, cytosolic calcium level, DNA fragmentation, and exposure of phosphatidylserine, were also evaluated. Despite the activity against *L. infantum* promastigotes in suspension, sugiol solubility presented an issue in the testing against intracellular amastigotes. In order to overcome this, we evaluated the capacity of YCWPs in delivering sugiol to macrophages directly. *In vitro* assays demonstrated that YCWPs were successfully engulfed by *L. infantum*-infected macrophages, and sugiol showed antileishmanial activity after release from the YCWPs in the target cell.

In summary, the sugiol antileishmanial activity, which caused secondary necrosis in *L. infantum* promastigotes, motivated us to use the YCWPs as a carrier system for this water-insoluble molecule to test against the *L. infantum* intracellular forms, which gave promising results. Therefore, we highlight this as a promising approach to cheaply and efficiently deliver sugiol and other insoluble compounds to treat leishmaniasis.

## Materials and Methods

### Isolation of Sugiol From *Cupressus lusitanica* Tree Bark

Sugiol was provided by the Chemistry Department of the Federal University of São Carlos. Briefly, *Cupressus lusitanica* barks were collected, dried at room temperature, and milled. The dried vegetal material was cold extracted 4 times with dichloromethane, and the extract was dried on a rotary evaporator, followed by size exclusion chromatography (h = 30.0 cm, Φ = 10.0 cm, silica gel 70–230 mesh) using hexane:ethyl acetate (90:10) as the elution system. This fraction was purified by column chromatography (h = 25 cm, Φ = 5 cm, silica gel size 230–400 mesh). The compound obtained after elution using hexane:ethyl acetate (98:02 and 96:04) was identified as sugiol by nuclear magnetic resonance spectroscopy (NMR).

### *In vitro* Activity of Free Sugiol Against *L. infantum*

*Leishmania infantum* MON-1 (MCAN/GR/82/LEM497) promastigotes were maintained in RPMI 1640 medium (Roswell Park Memory Institute – Gibco®) supplemented with 10% inactivated fetal calf serum (FCS - Invitrogen®) and incubated at 25°C. Parasites in the log phase of growth were placed in a 24-well microplate at a concentration of 1 ×10^6^ promastigotes/mL in the presence of increasing concentrations of DMSO-solubilized sugiol (0.15, 0.3, 1.5, 3.0, 15.0, and 30.0 μg/mL). The DMSO concentration never exceeded 0.003% (v/v) and did not interfere with parasite growth. Promastigotes were counted on a hemocytometer after 24, 48, and 72 h of incubation at 25°C. The IC_50_ or the concentration able to inhibit 50% of the growth compared to control was obtained by plotting the concentration of the compounds vs. the parasites inhibition percentage. Different concentrations (0.5, 5, 10, and 50 μg/mL) of Miltefosine were used as a positive control.

In order to evaluate sugiol activity against intracellular amastigotes, intraperitoneal macrophages were harvested from female BALB/c mice by injection of cold phosphate-saline buffer (PBS) plus 3% FCS. After centrifugation, cells were resuspended in RPMI medium and 5 ×10^5^ macrophages/mL were added per well of a 24-well microplate containing a glass coverslip, then incubated for 2 h at 37°C and 5% CO_2_ atmosphere for cellular adhesion. The non-adherent cells were washed off and adhered macrophages were infected with *L. infantum* promastigotes in the late log phase of growth (6–7 days in culture), at a ratio of 7 parasites:1 macrophage, for 4 h at 34°C and 5% CO_2_ atmosphere, followed by the addition of the drugs (Kaplum et al., 2016). Sugiol was tested at 0.9, 3.0, 9.0, and 18.0 μg/mL. Miltefosine (0.05, 0.5, and 5.0 μg/mL) was used as a control. After 48 h, the glass coverslips were fixed with methanol for 10 min and stained with 10% Giemsa stain for 40 min. On a light microscope, 200 macrophages per coverslip were evaluated to determine the number of macrophages infected and the number of amastigotes within each infected macrophage. The survival index (SI = infected cells percentage × amastigote average per infected macrophage) was determined. Survival index of amastigotes from untreated infected macrophages (negative control – NC) was considered as 100% of survival for the purpose of IC_50_ calculation.

### Cytotoxicity Assay

Macrophages were harvested from mice according to the description above. Macrophages were seeded in a 96-well microplate at 1 ×10^6^ cells/mL and incubated for 4 h at 37°C and 5% CO_2_ to allow cell adhesion. Increasing concentrations of DMSO-solubilized sugiol (10–300 μg/mL) were added and incubated under the same conditions for 48 h. The DMSO concentration (v/v) in all treatments, including in the negative control, never exceeded 0.03%, which is considered non-toxic for mammalian cells. (Chen et al., 2005; Mullol et al., 2006; Elisia et al., 2016; de Abreu Costa et al., 2017). For the evaluation of macrophage viability, 50 μL MTT (2 mg/mL - Invitrogen®) was added to each well and incubated for 4 h in the absence of light. Mitochondrial dehydrogenases from viable macrophages reduce MTT, resulting in purple crystals of formazan. After solubilizing formazan crystals in DMSO, the absorbance was measured on a microplate reader (BIO-TEK Synergy HT) at 570 nm (Mosmann, 1983). A dose-response curve was created and enabled the calculation of the cytotoxicity concentration that reduced the absorbance value by 50% when compared to the untreated negative control, represented as the CC_50_.

### Ethics Statement

This study was approved and performed following the recommendations of the Ethical Committee of Animal Use of State University of Maringá (protocol n° 1323011116/2017).

### Morphological and Ultrastructural Analysis

*L. infantum* promastigotes (1 ×10^6^ parasites/mL) in the log phase of growth were treated with sugiol (4.1 and 30.0 μg/mL), incubated for 72 h at 25°C, then fixed in 2.5% glutaraldehyde in 0.1 M sodium cacodylate buffer, pH 7.2 (Electron Microscopy Sciences—EMS®) for 24 h. Parasites were adhered to poly-L-lysine (Sigma-Aldrich®) coated glass slides. Samples were dehydrated with increasing concentrations of ethanol (30–100%), followed by critical point drying in CO_2_ (Baltec SCD-030) to remove any water trace. Samples were then mounted on a stub and coated with gold. The analysis was carried out on FEI-Quanta 250 scanning electron microscope (SEM).

For ultrastructural analysis, after 72 h of incubation at 25°C, promastigotes treated with sugiol (4.1 μg/mL) were centrifuged at 2,000 × g for 10 min, and the cell pellet was fixed with 2.5% glutaraldehyde in 0.1 M sodium cacodylate buffer (pH 7.2) for 3 h. Post-fixation was performed using 1% osmium tetroxide (Electron Microscopy Sciences—EMS®) for 1 h, parasites were washed in the same buffer then contrasted in 1% aqueous uranyl acetate (Electron Microscopy Sciences—EMS®) for 1 h. Samples were dehydrated in a graded acetone series (70–100%) and embedded in 2% molten agar. Next, cell pellets were re-dehydrated in acetone (30–100%), impregnated, and included in epoxy resin (EPON^TM^). Ultrathin sections were mounted on copper grids and stained with 0.2% lead citrate for 7 min. Analysis was performed on a FEI-Tecnai G2 Spirit Bio Twin microscope at 100 kV.

### Size Analysis of *L. infantum* Promastigotes

Parasite suspensions (4 ×10^6^ promastigotes/mL) were treated with different concentrations of sugiol: 4.1, 15.0, and 30.0 μg/mL, and incubated for 48 h. Samples were washed with 0.01 M PBS and resuspended in 500 μL PBS. Miltefosine (Sigma-Aldrich®) at 16.0 μg/mL was used as a positive control. Miltefosine is a drug known for inducing apoptosis-like death, causing cell size reduction (Paris et al., 2004; Scariot et al., 2017; Basmaciyan et al., 2018). Promastigotes size was measured on a BD FACSCalibur flow cytometer based on FSC and SSC patterns of the population and 10,000 events were analyzed by CellQuest Pro software. Median value analysis was performed and a histogram was generated, considering FSC as a function of cell size.

### Detection of Phosphatidylserine Exposure

Parasite suspensions (4 ×10^6^ promastigotes/mL) were treated with 4.1, 15.0, and 30.0 μg/mL sugiol and 16.0 μg/mL Miltefosine as the positive control (Paris et al., 2004; Scariot et al., 2017; Basmaciyan et al., 2018). ApopNexin Annexin V/FITC Apoptosis Kit (Merck®) was used to verify the phosphatidylserine (PS) exposure after 48 h of treatment. Briefly, the samples were washed with PBS and resuspended in 200 μL 1x binding buffer (140 mM NaCl; 5 mM CaCl_2_; 10 mM HEPES-Na; pH 7.4), followed by the addition of 3 μL Annexin V-FITC and 2 μL propidium iodide (PI). After 15 min in the dark at room temperature, the samples were analyzed on a BD FACSCalibur flow cytometer (10,000 events), using FL-1 and FL-3 as parameters, in accordance with the manufacturer's instructions. FITC fluorescence can be detected if PS is exposing the specific binding site for annexin V, which is a sign of an apoptotic process developing (Zwaal et al., 2005). For analysis, annexin V–PI– were considered viable parasites; annexin V+PI–, early apoptotic parasites; and annexin V+PI+, parasites in late apoptosis/secondary necrosis (Efstathiou et al., 2014; Antinarelli et al., 2018; Argüello-García et al., 2018; Vishwakarma et al., 2018; Keighobadi et al., 2019; Zahedifard et al., 2019).

### Quantitative Analysis of Autophagic Vacuoles

The quantification of autophagic vacuoles was carried out using the MDC (monodansylcadaverine – Sigma-Aldrich®), an autofluorescent molecule. MDC accumulates in lipid-rich membranous compartments, such as autophagic vacuoles, and gets trapped in adjacent membranes (Vázquez and Colombo, 2009). Promastigotes (4 ×10^6^ cells/mL) were treated with 4.1, 15.0, and 21.0 μg/mL sugiol for 24, 48, and 72 h. For the positive control, promastigotes were submitted to the stress of nutrient scarcity by diluting the culture medium with PBS (Bootman et al., 2017). After washing the cells, MDC was added at a final concentration of 50 μM, and incubated for 60 min at 25°C. All treatments were also evaluated with wortmannin, a known inhibitor of the autophagic vacuole formation, at a final concentration of 1 mM (Blommaart et al., 1997). Fluorescence measurement was performed on a Victor X3 fluorometer at λ_ex_ = 380 nm and λ_em_ = 525 nm (Pincus et al., 1975).

### Analysis of Transmembrane Mitochondrial Potential

Mitochondrial membrane potential was measured through TMRE labeling. TMRE can permeate the cell and accumulate in the functional mitochondria emitting fluorescence. Mitochondrial potential dysfunction can increase or decrease the sequestration of TMRE (Crowley et al., 2016). *L. infantum* (4x10^6^ promastigotes/mL) were treated with 4.1, 15.0, and 21.0 μg/mL sugiol for 24, 48, and 72 h. Samples were washed, resuspended in saline solution (0.9% sodium chloride solution), and TMRE (Sigma-Aldrich®) was added at a final concentration of 0.25 μM. After 30 min in the dark, the samples were washed, and the measurement was performed on a Victor X3 fluorometer, using λ_ex_ = 495 nm and λ_em_ = 595 nm. As the positive control, CCCP (carbonyl cyanide 3-chlorophenylhydrazone – Sigma–Aldrich®) at 100 μM was applied at the end of each incubation (Chen et al., 2004).

### Generation of Reactive Oxygen Species

Parasite suspensions (4 ×10^6^ promastigotes/mL) were treated with 4.1, 15.0, 21.0, and 30.0 μg/mL sugiol. Parasites incubated with 0.25 mM hydrogen peroxide (H_2_O_2_) were used as a positive control. After incubation, parasites were washed with PBS, and 2.5 μL H_2_DCFDA (2′,7′-dichlorodihydrofluorescein diacetate – Sigma-Aldrich®) at 1 mg/mL was added and incubated for 45 min at room temperature in the absence of light. This non-fluorescent chemical dye is metabolized by intracellular esterase followed by the oxidation by ROS, generating the fluorescent 2',7'-dichlorofluorescein molecule. Fluorescence intensity was quantified on a BIO-TEK Synergy HT reader plate at λ_ex_ = 488 nm and λ_em_ = 530 nm (Shukla et al., 2012).

### Evaluation of Lipid Peroxidation

Parasite suspensions (4 ×10^6^ promastigotes/mL) were treated with 4.1, 15.0, 21.0, and 30.0 μg/mL sugiol and incubated for 24, 48, and 72 h. As a positive control, cells were treated with 2.0 μM H_2_O_2_ for the same time. After washing and resuspending the cells in PBS, DPPP (1,3-Bis(diphenylphosphino)propane - Sigma-Aldrich®) was added at a final concentration of 50 μM. The samples were incubated for 15 min at 25°C and the fluorescence measured on a Victor X3 fluorometer at λ_ex_ = 355 nm and λ_em_ = 460 nm. DPPP is a non-fluorescent molecule which reacts with fatty acids, phosphatidylcholine, and triacylglycerol hydroperoxides, resulting in a highly fluorescent molecule (Akasaka et al., 1987; Takahashi et al., 2001).

### Measurement of the Intracellular Calcium Level

Fluo-4AM (Thermo Fisher Scientific®) was added to 4 ×10^6^ promastigotes/mL at a final concentration of 5 μM, according to manufacturer's instructions. Fluo-4AM permeant molecules react with intracellular esterase making the site available to bind free calcium (Ca^2+^) and, consequently, emitting fluorescence. After 60 min incubation at 25°C, different concentrations of sugiol (4.1, 15.0, 21.0, and 30.0 μg/mL) were added to the parasites. The fluorescence measurements were read immediately after the treatment and at 10 min intervals on a BIO-TEK Synergy HT reader plate at λ_ex_ = 495 nm and λ_em_ = 506 nm. Calcium concentration was determined by the following equation: [Ca^2+^] = K_d_ (F-F_min_)/(F_max_-F), where K_d_ = 345 nM. For the maximum fluorescence (F_max_), where calcium overload led to the total fluorescence of the label, 80 mM CaCl_2_ plus 40 μM digitonin were added. For the minimum fluorescence (F_min_), 45 mM EGTA was used. EGTA is able to chelate all free Ca^2+^ keeping the molecule in the non-fluorescent state (Dolai et al., 2011). Parasites treated with 8 mM H_2_O_2_ were used as a positive control (Das et al., 2001).

### Detection of DNA Fragmentation

DNA fragmentation was evaluated by flow cytometry using PI (Sigma-Aldrich®) as the fluorescent label. Promastigotes (4 ×10^6^ cells/mL) in the log phase of growth were treated with sugiol at 4.1, 15.0, 21.0, and 30.0 μg/mL for 48 h. Miltefosine at 16.0 μg/mL was used as a positive control (Bhalla et al., 1993; Verma and Dey, 2004). Samples were fixed in 70% methanol at −20°C for 48 h. Next, 10 μg/mL PI and 10 μg/mL RNase A was added, and the samples incubated for 45 min at 37°C in the dark. After this period, PI fluorescence was analyzed on a BD FACSCalibur flow cytometer (10,000 events), using FL-2 as the parameter. The data were acquired on CellQuest Pro software. PI fluorescence enables the measurement of the DNA content during each phase of mitosis. Small DNA fragments, which typically occur during the apoptotic process, were lost during the fixation process, therefore, the DNA content was lower in these cells, causing an increase in the number of cells in the cell cycle sub-G0/G1 phase. Cell cycle data were analyzed and processed on a ModFit LT^TM^. Additionally, DNA fragmentation was assayed by agarose gel electrophoresis. For this, DNA extraction from promastigotes was carried out using the NZY Tissue gDNA Isolation Kit, following the manufacturer's instructions. Extracted DNA was mixed with bromophenol blue and applied to a 1.5% agarose gel in Tris-acetate-EDTA (TAE) buffer (40 mM Tris-base, 20 mM acetic acid, 1 mM EDTA in deionized H_2_O; pH 8.4) containing ethidium bromide (0.5 μg/mL). The molecular-weight size marker used was 1 kb Plus DNA Ladder (Thermo Fisher Scientific). After running the samples, the gel was photographed (UVITEC-UVISAVE, Alfagene) on an ultraviolet light.

### Preparation of YCWPs and Determination of Sugiol Encapsulation Efficiency

Due to weak solubility in aqueous medium, sugiol was entrapped in YCWPs to improve the delivery of this drug into macrophages. Yeast cell wall particles, rich in β-1,3-D-glucan, were obtained from *Saccharomyces cerevisiae*. Briefly, *S. cerevisiae* (20 g) from baker's yeast (Mauripan®) was suspended in 200 mL 1 M NaOH, and stirred under heating (85°C) for 60 min. The insoluble material in the cell walls was recovered by centrifugation at 2,000 × g. These steps were repeated three times, with the last step stirring for 10 min. Finally, the material was extracted in deionized water (pH 4.5), at 75°C for 60 min. Insoluble material was recovered by centrifugation, washed three times in water, isopropanol, and acetone. After drying at room temperature, the cell wall particles were free of any intracellular content, proteins, or chitin (Soto and Ostroff, 2008; Pan et al., 2015).

In order to entrap sugiol, 100 μL of a sugiol acetone solution (500 μg/mL) was mixed with 10 mg YCWPs, incubated at −20°C for 2 h. Next, samples were dried at room temperature. To promote a high accumulation of sugiol within the YCWPs, up to 5 cycles of encapsulation were performed. The encapsulation efficiency after each cycle was determined after washing the samples in acetone to remove all unincorporated drug. The supernatant was recovered and the concentration of unincorporated sugiol was measured on the spectrophotometer (Shimadzu UV1603) at 207 nm. Thus, it was possible to determine the efficiency of encapsulation (EE) by this indirect method using a standard curve. The results from 3 independent experiments are shown, expressed as the mean ± standard deviation.

### Characterization of YCWPs and YCWPs Containing Sugiol

#### Electron Microscopy (EM)

Scanning electron microscopy (SEM) was carried out to analyze the morphology and topography of YCWPs. The dehydrated sample was attached on the stub and coated with gold on the Bal-Tec SCD 050 Sputter Coater. After homogeneously depositing this conducting layer of electrons, the samples were ready to be visualized on the Shimadzu SS-550 scanning electron microscope.

For transmission electron microscopy (TEM), a 5 μL of suspension of either empty YCWPs or YCWPs containing sugiol (YCWPs+sugiol) at 2 mg/mL was placed on the copper grid covered with formvar/carbon film (300 mesh—Electron Microscopy Sciences—EMS®). After 2 min at room temperature, the hydrated YCWPs were analyzed on the FEI – Tecnai G2 Spirit Bio Twin transmission electron microscope.

#### Size and Zeta Potential Determination

Zeta potential, size, and polydispersity index (PDI) were measured using a Delsa™ Nano C Particle Analyzer (Beckman Coulter, Madrid, ES) for empty and loaded YCWP from three different production batches. The values for zeta potential were calculated as mean electrophoretic mobility values using Smoluchowski's equation. Particle size was determined by dynamic light scattering (DLS) at a 165° angle. All the analyzes were performed at 25°C. Each measurement was repeated 3 times, and the results are expressed as the mean ± standard deviation.

#### FT-IR Analysis

Fourier transform infrared (FT-IR) spectrum could be summarized as the correlation between the electromagnetic radiation and the frequencies from the vibration of the specific chemical bond. FT-IR was used in this study to characterize the empty YCWPs and YCWPs+sugiol by transmittance measurement. For this, potassium bromide (KBr) disks containing ~5% sugiol, empty YCWPs or YCWPs+sugiol were prepared by application of high pressure on a digital hydraulic press PIKE CrushIR. Data were acquired by FT-IR spectroscopy (Spectrum 400 FT-IR/FT-NIR – Perkin-Elmer) in the spectral range between 450 and 4,000 cm^−1^ and a resolution of 2 cm^−1^.

### The Biological Activity of YCWPs and YCWPs+sugiol Against *L. infantum* Parasites and Macrophages

A suspension of 1 ×10^6^ *L. infantum* promastigotes/mL in the log phase of growth was added to the wells of a 24-well-microplate in the presence of YCWPs+sugiol. YCWPs (1 mg) contained different entrapped sugiol mass (1.5, 3.7, 6.5, and 7.0 μg), as determined at the end of each entrapment cycle. The same procedure was performed to test different concentrations of empty YCWPs (10, 5, 1, and 0.5 mg/mL). The IC_50_ was determined after 48 h of incubation at 25°C.

Assays for antileishmanial activity against *L. infantum* amastigotes was performed using intracellular parasites in peritoneal macrophages, as previously described (section *In vitro* activity of free sugiol against *L. infantum*). The final concentration of YCWPs in the assays against amastigotes was standardized as 1 mg/mL. Therefore, the infected macrophages were treated with 1 mg/mL empty YCWPs and with 1 mg/mL loaded YCWPs, containing 1.5, 3.7, 6.5, and 7.0 μg/mL sugiol. By maintaining a fixed concentration of YCWPs, any contributing effect by the particle can be eliminated, making it possible to evaluate just the activity of the encapsulated sugiol. Using the determination of the survival index, as described above, it was possible to calculate the IC_50_ by constructing a dose-response curve. Optical microscopy enabled visual and quantitative verification of the reduction of amastigote numbers inside the macrophages due to the activity of YCWPs+sugiol.

Macrophage viability after treatment with sugiol, empty YCWPs, and YCWPs+sugiol was evaluated on peritoneal macrophages through the use of a colorimetric MTT assay (Mosmann, 1983). Briefly, 1x10^6^ peritoneal macrophages/mL were treated with increasing concentrations of empty YCWPs (0.5, 1, 5, and 10 mg/mL). Additionally, macrophages were treated with YCWPs+sugiol as follows: 10 mg/mL YCWPs containing 70 μg/mL sugiol; 5 mg/mL YCWPs containing 35 μg/mL sugiol; 1 mg/mL YCWPs containing 7 μg/mL sugiol; and 0.5 mg/mL YCWPs containing 3.5 μg/mL sugiol. Cell viability was measured by the reduction of MTT, analyzed by spectrophotometry at 570 nm. CC_50_ was determined by constructing a dose-response curve.

### Transmission Electron Microscopy Analysis of *L. infantum* Infected Macrophages

The presence of amastigotes and YCWPs within macrophages, as well as the inner organization of macrophages, were evaluated by TEM, with qualitative purposes. To minimize the experimental use of animals and in compliance with the recommendations of the animal use ethics committee, TEM samples were prepared using a macrophage cell line as opposed to the peritoneal macrophages, since a large number of mice would be necessary to obtain enough macrophages for performing this technique. Thus, J774.A1 macrophages (2.5 ×10^6^ cells) were infected with promastigotes in the late log phase of growth at a ratio of 10 promastigotes:1 macrophage. The cells were incubated at 34°C and 5% CO_2_ overnight, then washed with PBS to remove the non-internalized promastigotes. Infected macrophages were treated with empty YCWPs at 1 mg/mL, YCWPs+sugiol (1 mg/mL YCWPs containing 7 μg/mL sugiol), and free sugiol at 5.7 μg/mL, using 10 mL as the final volume. All these concentrations were responsible in causing ~50% of amastigote growth inhibition, as determined in the IC_50_ assays with infected macrophages. After 48 h, the cells were fixed in glutaraldehyde 2.5% in 0.1 M sodium cacodylate buffer for 24 h. The samples were post-fixed in 1% osmium tetroxide (OsO_4_) and 0.8% potassium ferrocyanide (Electron Microscopy Sciences—EMS®), and dehydrated in increasing concentrations of acetone (30–100%). EPON^TM^ epoxy resin was used to replace the acetone. At the end of the process, epoxy resin was polymerized at 60°C for 48 h. Samples were observed on a transmission electron microscope JEOL-JEM 1400 after obtaining nanometric cuts (60–70 nm) on an ultramicrotome, which were contrasted with 5% uranyl acetate and 2% lead citrate.

### Statistical Analysis

Numerical results were expressed as the mean of three experiments ± standard deviation (SD), and each experimental replicate was constituted by 4 biological replicates. Statistical analysis was performed using the GraphPad Prism software, and statistical significance was determined by one-way ANOVA test. Two-way ANOVA test was used to examine data with multiples variables, and Bonferroni's post-test evaluated the intergroup statistical difference.

## Results

### Free Sugiol Was Active Against *L. infantum* Promastigotes

*L. infantum* promastigote growth was verified after 24, 48, and 72 h of treatment with free sugiol (see Supplementary Material). The IC_50_ values were 10.6 ± 1.1 μg/mL, 5.5 ± 0.6 μg/mL, and 4.1 ± 0.2 μg/mL, respectively. Miltefosine was used as a positive control with IC_50_ values of 0.8 ± 0.1 μg/mL, 0.9 ± 0.2 μg/mL, and 1.1 ± 0.17 μg/mL after incubation for 24, 48, and 72 h, respectively.

### Free Sugiol Caused Morphological and Ultrastructural Changes in Promastigotes

**Figure 1:**
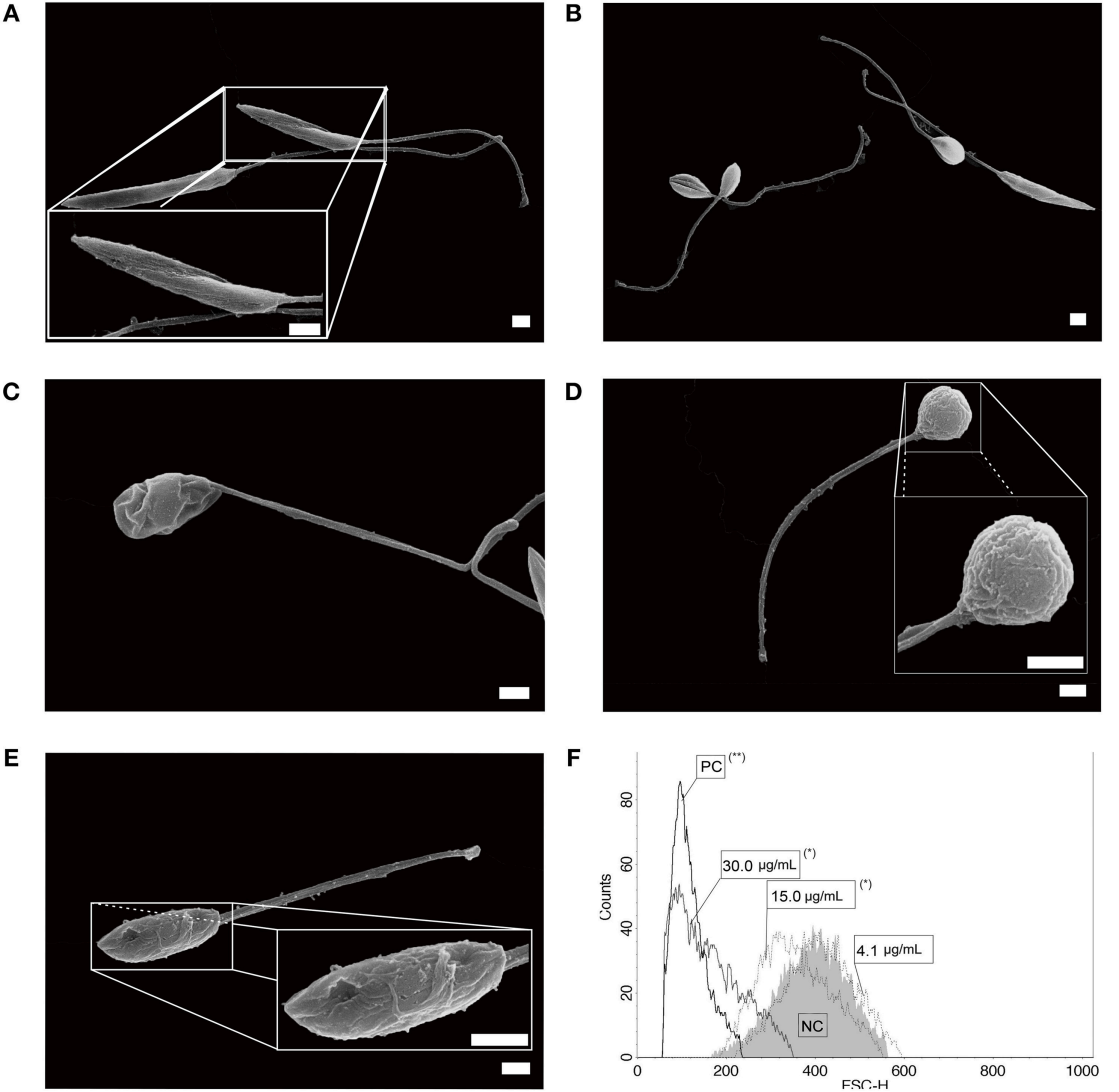
*L. infantum* promastigotes observed on SEM. **(A)** non-treated parasites; **(B,C)** parasites after 72 h of incubation with 4.1 μg/mL of sugiol, representing the IC_50_, and **(D,E)** with 30.0 μg/mL of sugiol (scale bar = 1 μm). Cell size was evaluated by flow cytometry after treatment with different concentrations of sugiol, according to the histogram **(F)**. Miltefosine at 16.0 μg/mL was used as positive control. *p <0.05 and **p <0.01, significant difference compared with negative control (NC) group.

**Figure 2:**
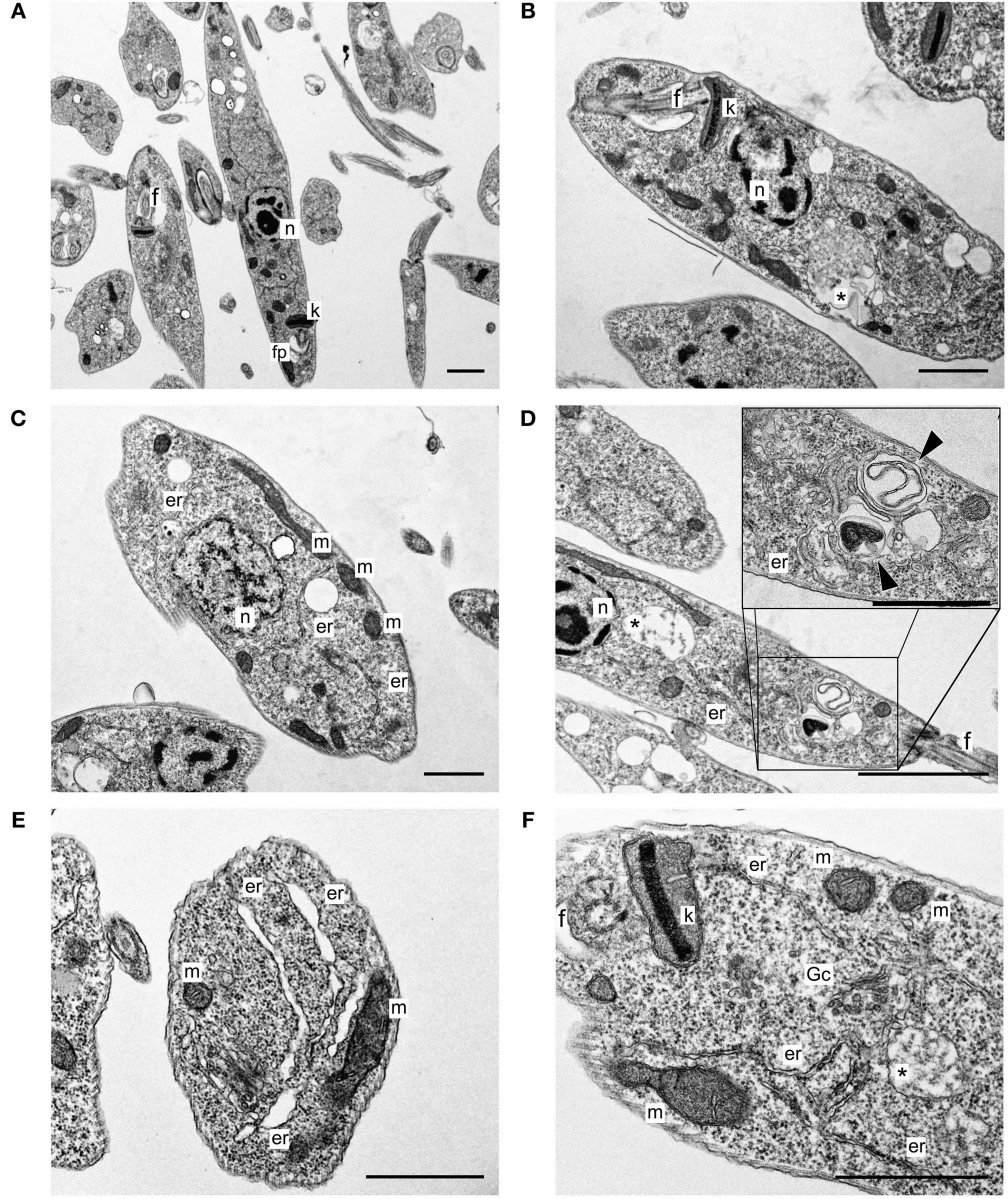
*L. infantum* promastigotes observed on TEM. **(A)** Non-treated parasites; **(B–F)** parasites after 72 h of treatment using 4.1 μg/mL of free sugiol. *n*, nucleus; k, kinetoplast; f, flagellum; fp, flagellar pocket; *autophagic vacuole; m, mitochondria; er, endoplasmic reticulum; Gc, Golgi complex; (head arrow) myelin-like figures (scale bar = 1 μm).

Typical untreated *L. infantum* promastigotes at log phase of growth had an elongated body, smooth cell membrane and a preserved anterior flagellum (Figure 1A). These morphological features were altered after treatment with free sugiol. Sugiol-treated parasites had a rounded body shape and exhibited cell surface roughness (Figures 1B–E). Despite the cell membrane alteration, leakage of intracellular contents was not observed (Figures 1C,E). A reduction in the size of the treated promastigotes could also be confirmed by the flow cytometry, which indicated significant cellular shrinkage (Figure 1F). TEM revealed well-preserved structures in untreated parasites (Figure 2A). However, after treatment with free sugiol, TEM micrographs showed the presence of autophagic vacuoles (Figures 2B,D,F), atypical nuclei (Figure 2C), and discrete mitochondrial swelling (Figure 2F). Disorganization of intracellular membranes, such as myelin-like figures (Figure 2D), derived from the swollen endoplasmic reticulum (ER) were also observed (Figure 2E).

### Free Sugiol Induced the Formation of Autophagic Vacuoles

**Figure 3:**
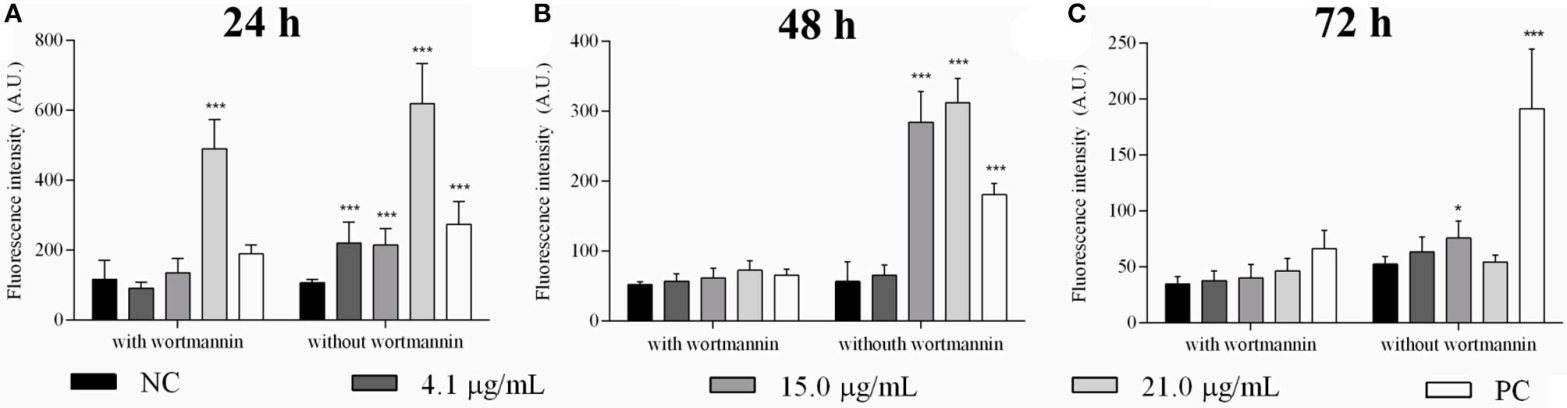
Detection of autophagic vacuoles in *L. infantum* promastigotes treated with free sugiol. Bars indicate the mean ± SD of MDC fluorescence used to detect autophagic vacuoles induced by different concentrations of sugiol after **(A)** 24 h, **(B)** 48 h, and **(C)** 72 h. Wortmannin, a known inhibitor of autophagic vacuole production, was used to ensure the MDC fluorescence specificity. Generation of autophagic vacuoles in promastigotes promoted by starvation was the strategy to obtain the positive control (PC). Fluorescence was measured in arbitrary units (A.U.) with Perkin Elmer Victor-X3 fluorometer. Three independent experiments were performed. *p <0.05 and ***p <0.001, significant difference in comparison to the negative control (NC) group.

Autophagic vacuoles were detected in promastigotes after 24, 48, and 72 h of sugiol treatment using MDC fluorescent labeling. A higher fluorescence intensity after 24 h of treatment indicated that sugiol promoted the formation of autophagic vacuoles by this time (Figure 3A). Incubation in the presence of wortmannin significantly inhibited the formation of autophagic vacuoles except in the treatment with the highest concentration of sugiol, 21.0 μg/mL, at 24 h, suggesting that MDC accumulated in vacuoles from another origin due to the intense damage caused by sugiol (Figure 3B). Increasing the concentration of free sugiol lead to increased autophagic vacuole formation at 24 h, but for all concentrations, the MDC fluorescence had decreased by 72 h of treatment. Sugiol at 15.0 μg/mL was the only treatment that generated autophagic vacuoles after 72 h of incubation (Figure 3C).

### Free Sugiol Induced Secondary Necrosis Death in *L. infantum* Promastigotes

**Figure 4:**
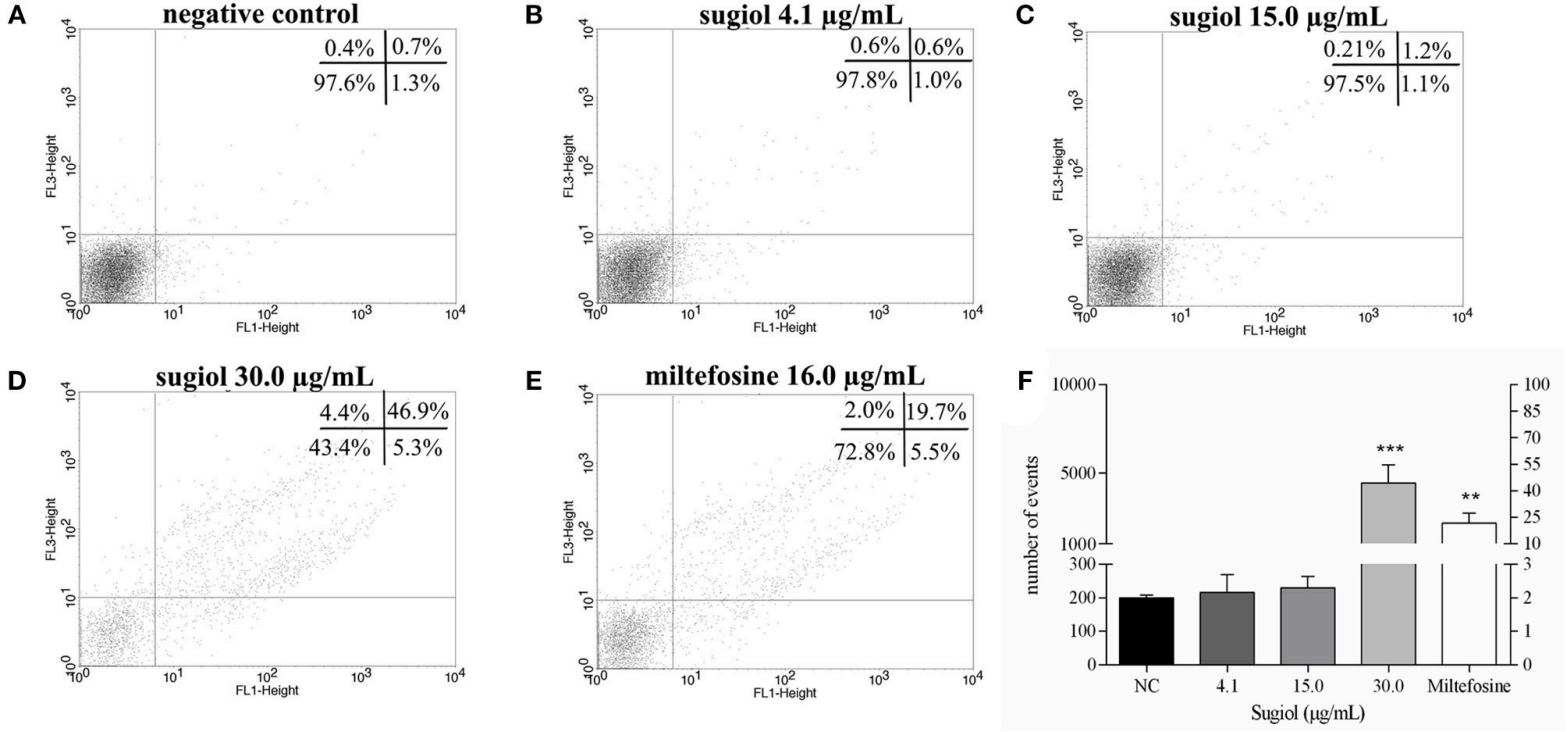
Exposure of phosphatidylserine and plasma membrane integrity detected on BD FACScalibur flow cytometer using Annexin V-FITC and PI. **(A)** Non-treated promastigotes and **(B–D)** treated with different concentrations of sugiol. **(E)** Miltefosine at 16.0 μg/mL was used as positive control. In each experimental replicate, 10,000 events were analyzed. Representative dot plot of three independent experiments was shown. The mean ± SD of the events and the corresponding percentage from Annexin V^+^ represented in upper (Annexin-V+PI+) and lower (Annexin-V+PI-) right quadrants in the dot plot is showed in the letter **(F)**. **p <0.01 and ***p <0.001, significant difference in comparison to the negative control (NC) group.

Promastigotes treated with sugiol were evaluated for the exposure of phosphatidylserine (PS) on the cell surface, a sign of apoptotic cell death. Untreated *L. infantum* promastigotes and promastigotes treated with 4.1 and 15.0 μg/mL sugiol for 48 h did not indicate PS exposure (Figures 4A–C). About 50.0% of the parasites presented as annexin V+PI+ after 48 h of treatment with the highest concentration of sugiol (30 μg/mL), suggesting the development of late apoptosis/secondary necrosis since the PI+ parasites also showed the loss of plasma membrane integrity (Figure 4D). In the same sample, a statistically non-significant population represented by only 5.3% of the promastigotes were annexin V+PI–, revealing those going through an early apoptotic process. Only 4.4% of the parasites presented signs of primary necrosis (annexin V-PI+) (Figure 4D). As expected, Miltefosine induced the late apoptotic/secondary necrosis process in promastigotes significantly (Figure 4E). Figure 4F represents the sum of early and late apoptotic/secondary necrosis events, showing that more than 50 and 20% of the promastigotes presented Annexin-V+ after treatment with 30.0 μg/mL of sugiol and 16.0 μg/mL of Miltefosine, respectively. Promastigotes treated with lower sugiol concentrations did not show significant fluorescence for Annexin-V in comparison to the untreated promastigotes.

**Figure 5:**
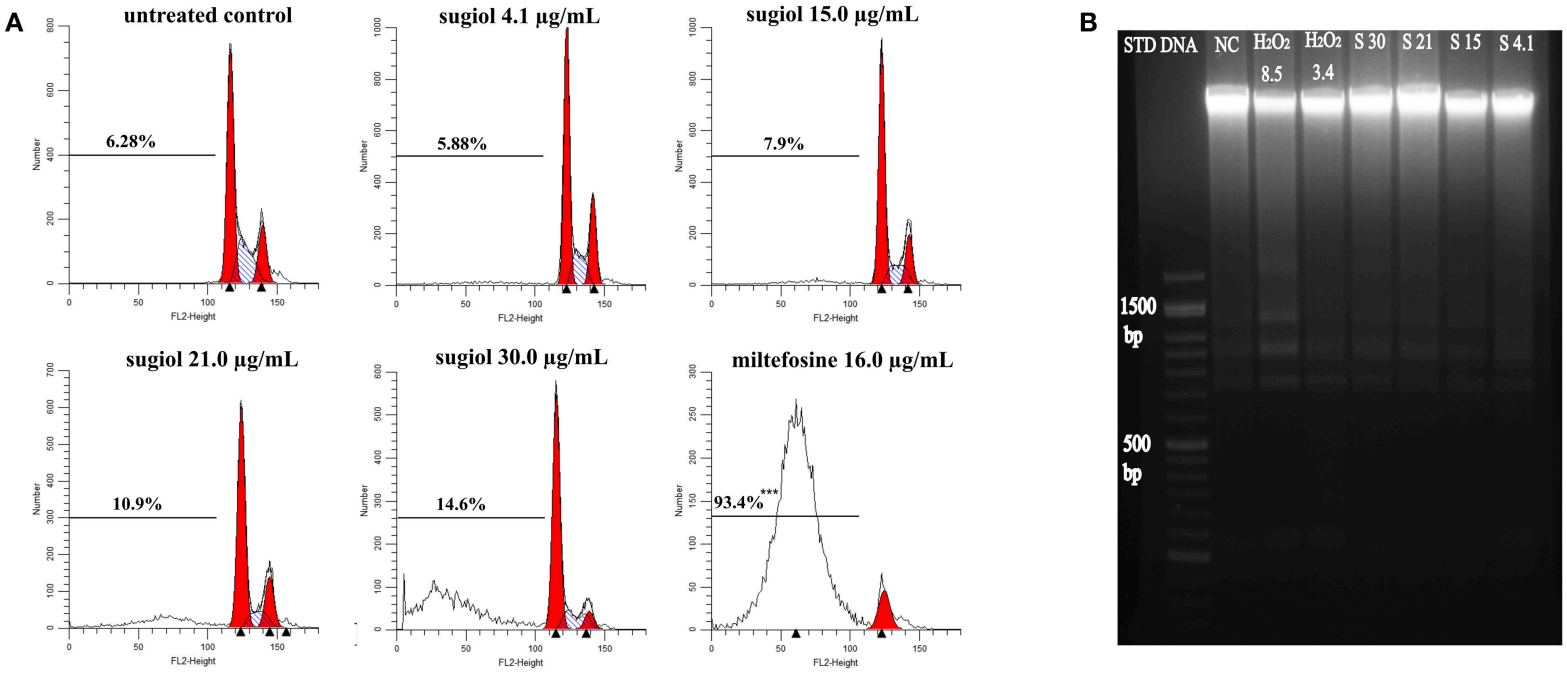
DNA integrity of *L. infantum* promastigotes treated with free sugiol. **(A)** DNA fragmentation detected by PI fluorescence on BD FACScalibur flow cytometer Typical representative histograms showed the percentage of sub G0/G1 phase, represented by the cells that lost small DNA fragments. Miltefosine at 16.0 μg/mL was used as positive control. Three independent experiments were performed and 10,000 events were analyzed in each one. ***p <0.001, significant difference compared to non-treated promastigotes. **(B)** Agarose gel electrophoresis of *L. infantum* promastigotes DNA. (STD DNA) molecular-weight size marker-−1 kb Plus DNA Ladder, (NC) untreated promastigotes, (H_2_O_2_ 8.5) hydrogen peroxide at 8.5 μg/mL was used as positive control; (S30), (S21), (S15), and (S4.1): promastigotes DNA after treatment with sugiol at 30.0, 21.0, 15.0, and 4.1 μg/mL, respectively.

Another feature of apoptotic cell death is DNA fragmentation, which was evaluated by another flow cytometry methodology using PI labeling and agarose gel electrophoresis. Flow cytometry indicated that sugiol did not induce DNA fragmentation in promastigotes because the percentage of hypodiploid promastigotes represented in the sub-G0/G1 cell cycle phase did not show a significant difference in comparison to the untreated control (Figure 5A). Corroborating this, agarose gel electrophoresis of DNA extracted from treated promastigotes showed no DNA fragmentation (Figure 5B). These results indicated that sugiol did not induce early apoptosis-like cell death in *L. infantum* promastigotes.

### Free Sugiol Increased the Cytosolic Calcium Level and Mitochondrial Disorders, Inducing Lipid Peroxidation in Promastigotes

**Figure 6:**
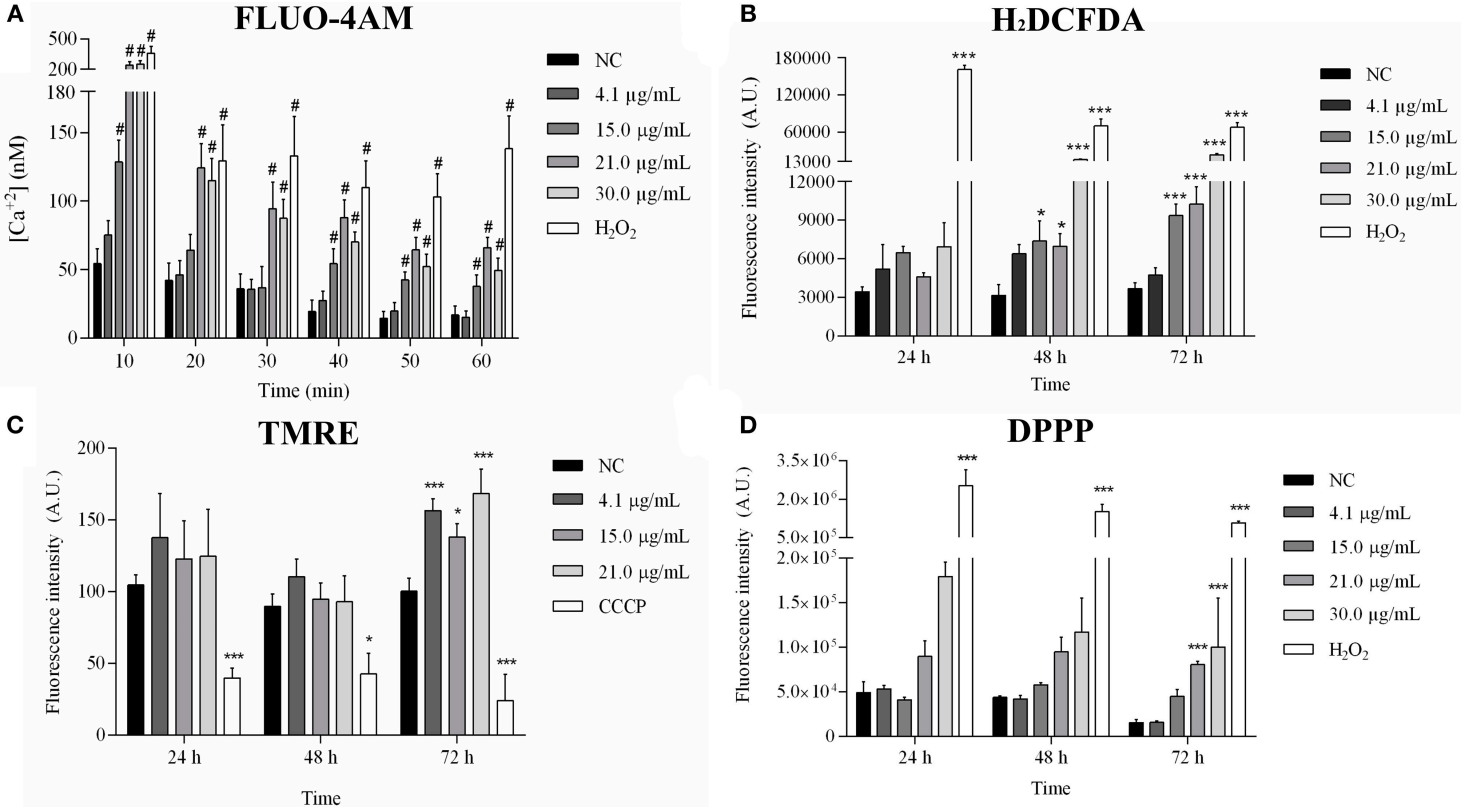
Mitochondrial profile analysis after 24, 48, and 72 h of exposition to sugiol. **(A)** Cytosolic calcium level in promastigotes treated with different concentrations of sugiol detected by FLUO-4AM molecule; **(B)** ROS production detection by H_2_DCFDA; **(C)** mitochondrial membrane potential evaluated using TMRE label; **(D)** detection of lipid peroxidation by DPPP label. Fluorescence was measured in arbitrary units (A.U.) using Perkin Elmer Victor-X3 fluorometer. H_2_O_2_ and CCCP were used as positive control. The results were expressed in the bar graphs as the mean ± SD of three independent experiments. *p <0.05 and ***^/#^*p* <0.001, significant difference compared to negative control (NC) group.

The cytosolic calcium level increased after treatment with 15.0, 21.0, and 30.0 μg/mL sugiol (Figure 6A). Reactive oxygen species (ROS) production increased significantly after 48 h of treatment, intensifying at 72 h (Figure 6B). TMRE labeling revealed hyperpolarization of mitochondrial membrane potential only at 72 h of treatment. As expected, CCCP, which was used as a positive control, caused mitochondrial membrane depolarization (Figure 6C). Lower concentrations of sugiol did not cause alterations in ROS production or in mitochondrial potential, and the lowest sugiol concentration (4.1 μg/mL) did not cause changes in calcium level either. At 72 h, there was a significant increase in lipid peroxidation in parasites treated with 21.0 and 30.0 μg/mL of free sugiol (Figure 6D). The positive control, H_2_O_2_, was able to induce lipid peroxidation, ROS production and enhanced the cytosolic calcium level at all evaluated times.

### Characterization of YCWPs and Sugiol Entrapment

**Figure 7:**
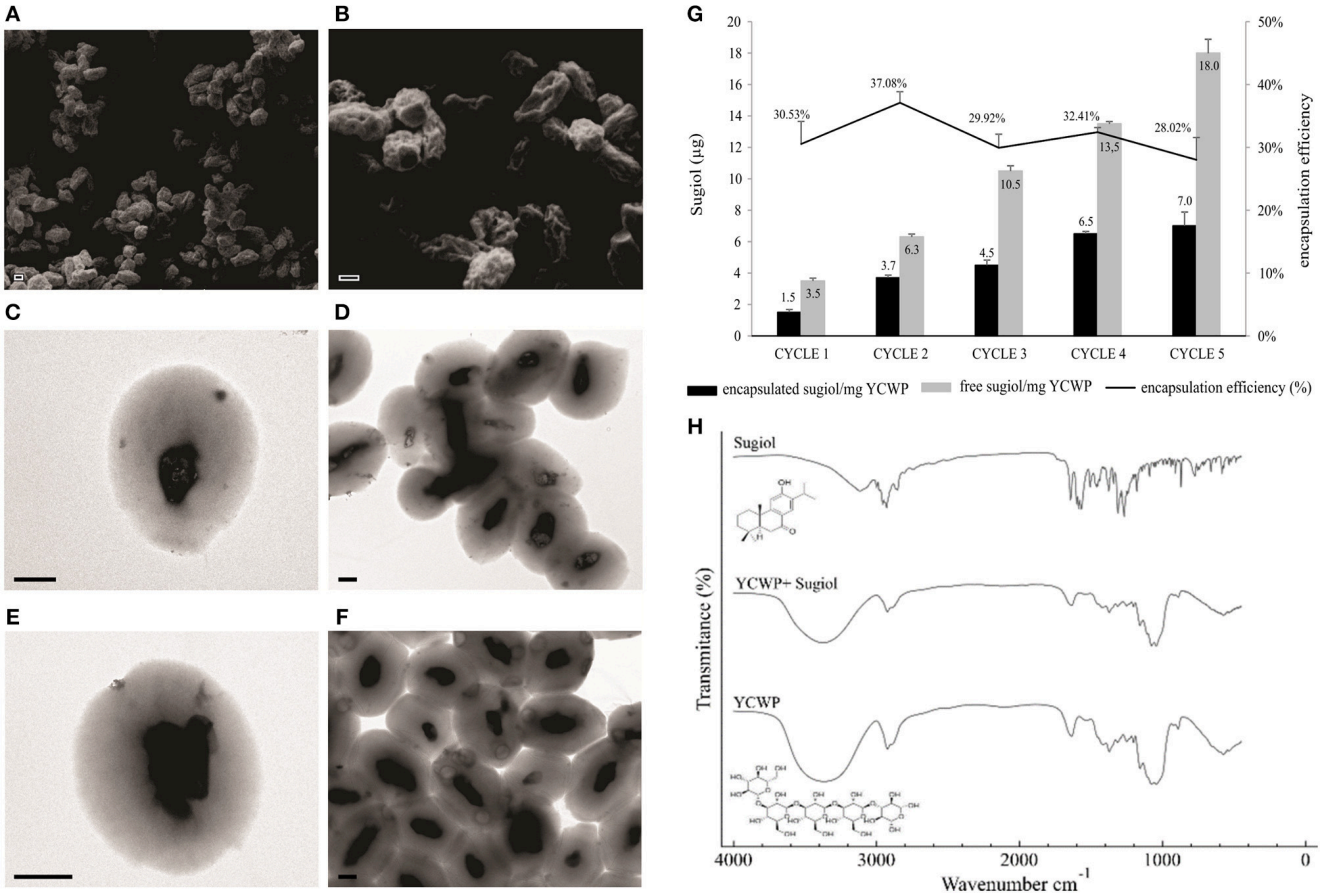
Characterization of YCWPs containing sugiol. **(A,B)** YCWPs visualized on SEM and TEM, being **(C,D)** empty YCWPs and **(E,F)** YCWPs+sugiol (scale bar = 1 μm). **(G)** Encapsulation efficiency after each cycle of encapsulation. The results were expressed as the mean ± SD of the mass per mg of YCWPs and percentage of sugiol inner and out of the particles from three independent experiments. Letter **(H)** shows the FT-IR spectrum obtained from empty YCWPs, YCWPs containing sugiol, and sugiol.

Despite the promising sugiol activity against *L. infantum* parasites, the compound solubility could be an issue for *in vivo* assays. For this reason, an alternative delivery method was designed for the delivery of sugiol into infected host cells, using YCWPs extracted from *S. cerevisiae* yeast. After isolation of the cell wall particles, free from intracellular content, and loading with sugiol, YCWPs were characterized by microscopy, FT-IR, and zeta potential analysis. SEM analysis showed dehydration of the particles, due to the acetone used during the isolation process (Figures 7A,B). The YCWP central cavity after hydration was observed on TEM micrographs, delimited by a typical thick cell wall structure (Figures 7C–F). Sugiol was entrapped in YCWPs by simple diffusion. Empty YCWPs and YCWPs+sugiol had sizes of 4.2 ± 0.3 and 3.8 ± 0.7 μm, respectively. Empty YCWPs showed neutral zeta potential of−5.0±0.8 before entrapment cycles. However, after an equal time of exposure to acetone during the entrapment cycles, empty and sugiol-loaded YCWPs exhibited similar negative zeta potential, represented by−15.5 ± 0.7 and−12.3 ± 0.7, respectively.

Sugiol encapsulation efficiency was on average 31% per cycle (Figure 7G). The maximum sugiol encapsulated mass was 7 μg per mg of YCWPs, at the fifth cycle. FT-IR spectroscopy analysis showed a typical spectrum of β-1,3-D-glucan for the empty YCWPs and YCWPs+sugiol. The absence of a sugiol molecule signal in the spectrum for the YCWPs containing sugiol suggested that the washing process was successful and no sugiol residues were detected outside the particle (Figure 7H).

### Free Sugiol, Empty YCWPs, and YCWPs+Sugiol Reduced the Number of Intracellular Amastigotes Causing Low to Moderate Toxic Effects in Macrophages

**Figure 8:**
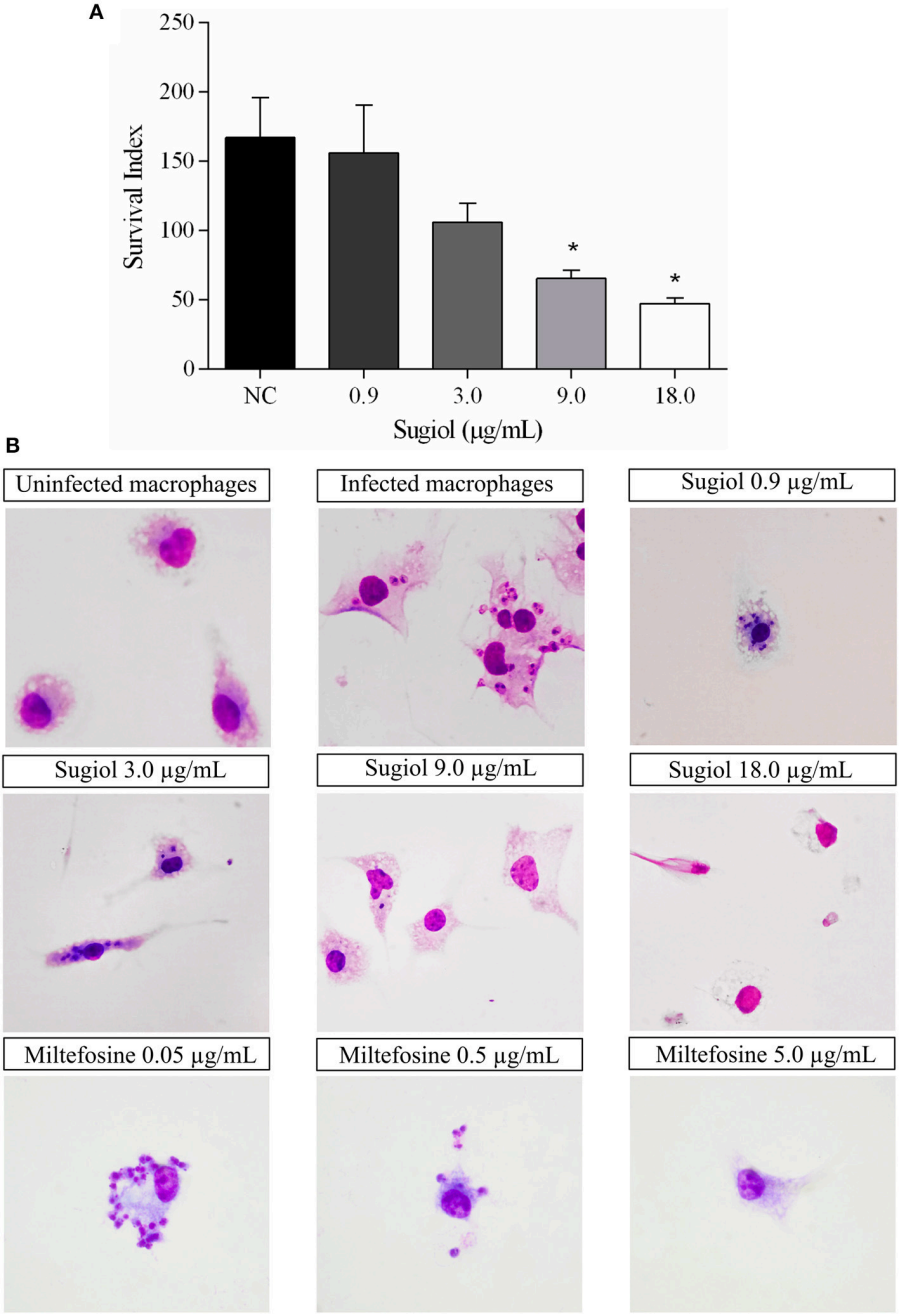
Sugiol activity against *L. infantum* amastigote. Amastigote survival index within **(A)** untreated infected macrophages (NC) and after treatment with different concentrations of sugiol for 48 h. Three independent experiments were performed and the results were expressed as the mean ± SD. *p <0.05, significant difference compared to negative control (NC) group. Letter **(B)** shows the optical microscopy of untreated and treated infected macrophages. Miltefosine was used as a positive control. Magnification: 1000x.

After 48 h of treatment, free sugiol presented an IC_50_ of 5.7 ± 0.4 μg/mL against intracellular *L. infantum* amastigotes. The amastigote survival index inside the macrophages was dose-dependent since the survival of the amastigotes decreased as the sugiol concentration increased (Figure 8A). Optical microscopy images corroborated this, showing that both the number of amastigotes and total number of infected macrophages was reduced (Figure 8B). Moreover, free sugiol cytotoxicity against the peritoneal macrophages suggested only a moderate toxic effect, with a CC_50_ of 80.5 ± 7.8 μg/mL. The selectivity index (SI)—the ratio between CC_50_ and IC_50_–showed that free sugiol against amastigotes was 14.1-fold more active compared to the effect on the macrophages. As an antileishmanial activity control, Miltefosine was used, presenting an IC_50_ of 0.11 ± 0.02 μg/mL against the parasites and a CC_50_ of 8.3± 0.2 μg/mL against peritoneal macrophages. However, it was still 75.4-fold more active against the parasite than the macrophage (Table 1).

**Table 1:** Antileishmanial activity (IC_50_) and cytotoxicity (CC_50_) from empty YCWPs and YCWPs+sugiol.

	**48 h**			
	**μg/mL ± SD**			
	**Sugiol**	**YCWPs**	**YCWPs+sugiol**	**Miltefosine**
CC_50_	80.5 ± 7.8	>10,000	>70^a^	8.3± 0.2
IC_50_ promastigote	5.5 ± 0.6	>10,000	>70^a^	0.9 ± 0.2
IC_50_ amastigote	5.7 ± 0.4	1,000 ± 0.1	6.8 ± 0.1^b^/ <1.5^c^	0.11 ± 0.02
SI^d^	14.1	>10	>10.3^b^/>46.7^c^	75.4

a *Mass of sugiol entrapped in 10,000 μg of YCWPs*.

b *IC_50_ in relation to the infect macrophages treated with empty YCWPs*.

c *IC_50_ in relation to the untreated control*.

d *Selectivity index considering the IC_50_ for amastigotes. Three independent experiments were performed. The results were expressed as the mean ± standard deviation (SD)*.

**Figure 9:**
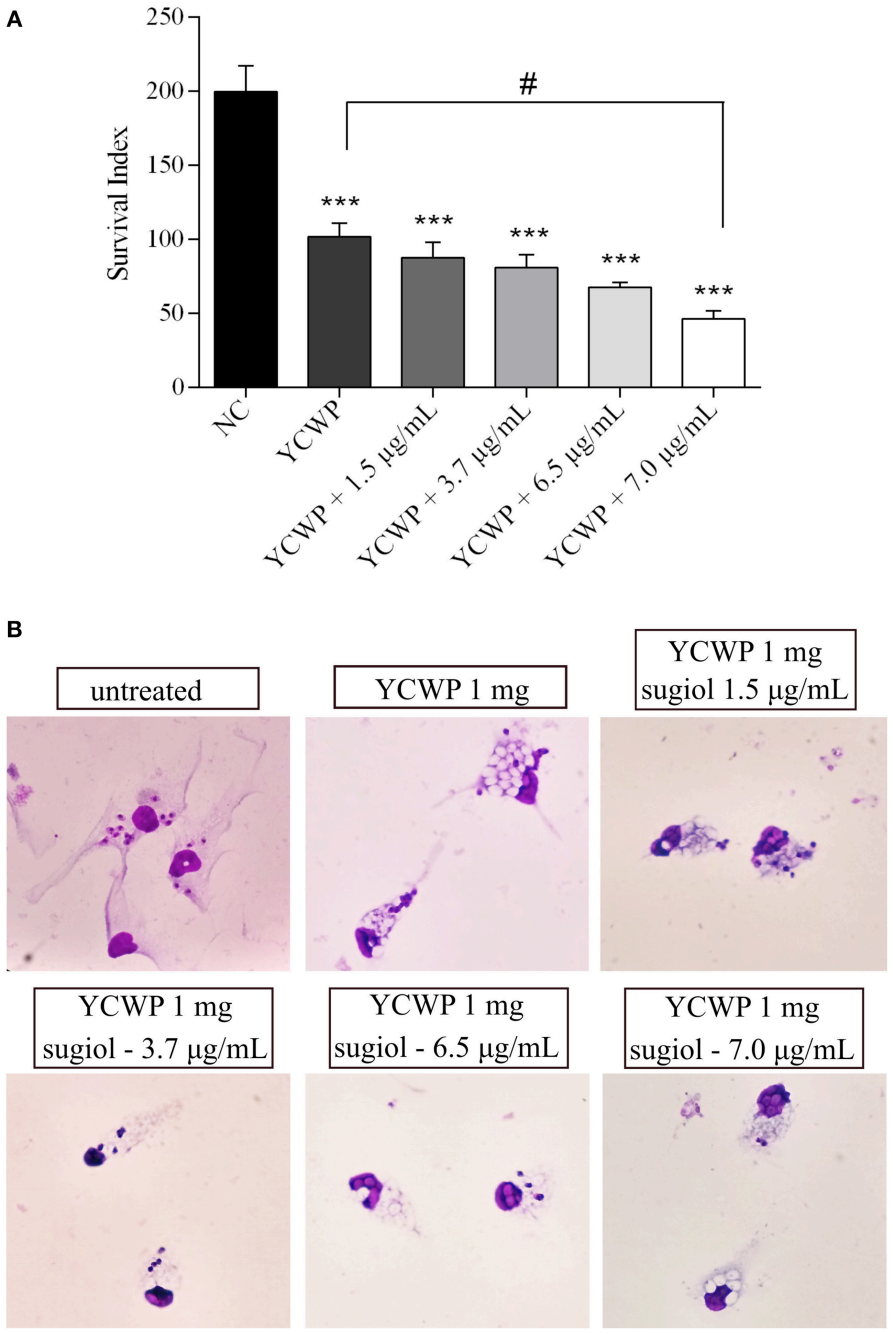
**(A)** Amastigote survival index within untreated macrophages (NC) and treated with 1 mg/mL of empty YCWPs and 1 mg/mL YCWPs containing different concentrations of sugiol. Three independent experiments were performed and the results were expressed as the mean ± SD. ***p <0.001, significant difference compared to negative control (NC) group; ^#^*p* <0.05, significant difference from YCWPs group. **(B)** Optical microscopy from untreated and treated infected macrophages. Magnification: 1000x.

Empty YCWPs at 1 mg/mL caused a 50.8% reduction in the number of amastigotes in infected macrophages, in comparison to the number of amastigotes in untreated infected macrophages. Entrapped sugiol acted in a concentration-dependent manner, as shown by the amastigote survival index in Figure 9A. To evaluate the effect of YCWPs and sugiol together, the survival index of an untreated control was considered in the IC_50_ calculation (Table 1). This showed that 1 mg/mL YCWPs containing 1.5 μg/mL sugiol inhibited 58.7 ± 0.1% of the amastigote growth. However, to suppress the interference of the intrinsic activity of YCWPs, the IC_50_ of YCWPs+sugiol was also determined considering the amastigote survival index in infected macrophages treated with empty YCWPs. In this case, the entrapped sugiol showed an IC_50_ = 6.8 ± 0.1 μg/mL. The reduction in amastigote number could be observed by optical microscopy, and signs of cytotoxicity after treatment with empty and sugiol-loaded YCWPs were not revealed. As YCWPs cannot be stained by Giemsa, the intense cytoplasmic vacuolization observed is due to the presence of YCWPs in vacuoles (Figure 9B).

Empty YCWPs and YCWPs+sugiol were also evaluated regarding the activity against *L. infantum* promastigotes. Interestingly, empty YCWPs and YCWPs+sugiol did not affect promastigotes (Table 1), suggesting that the entrapped sugiol was not released in the culture medium. Thus, only the process of loaded-YCWPs entering the macrophages was able to release the sugiol.

### Entrapped and Free Sugiol Caused Ultrastructural Changes in Intracellular Amastigotes

**Figure 10:**
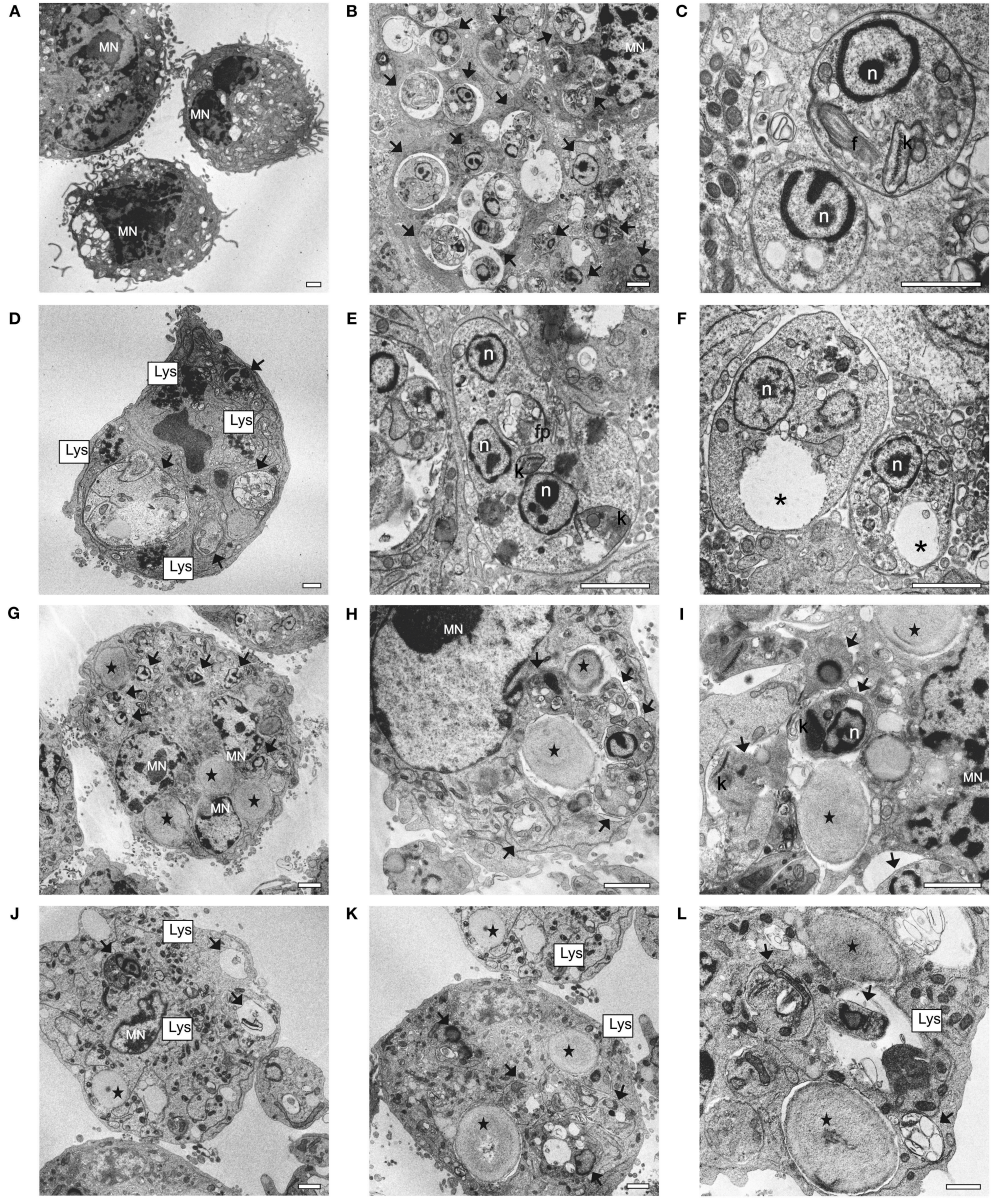
Ultrastructural changes in macrophages and *L. infantum* amastigotes observed by TEM analysis. **(A)** uninfected and untreated macrophages; **(B,C)** infected macrophages; **(D–F)** infected macrophages treated with 5.7 μg/mL of sugiol; **(G–I)** infected macrophages treated with empty YCWPs at 1 mg/mL; **(J–L)** infected macrophage treated with 1 mg/mL of YCWPs containing 7 μg/mL of sugiol. (MN) macrophage's nucleus; (black arrows) amastigotes; n, amastigote's nucleus; f, flagellum; k, kinetoplast; Lys, lysosomes; fp, flagellar pocket; *vacuole; (black star) YCWPs (scale bar = 1 μm).

TEM was performed to locate YCWPs inside the macrophages and observe the ultrastructural changes promoted by the action from empty and sugiol-loaded YCWPs on both the parasites and macrophages. Firstly, the ultrastructure of a typical uninfected macrophage was observed (Figure 10A). In the untreated infected control, single amastigotes were found within the parasitophorous vacuoles of macrophages (Figures 10B,C). Amastigotes showed a typical ultrastructural organization: rounded body, internalized flagella, with the nucleus and kinetoplast preserved (Figures 10B,C). For comparison, ultrastructural changes in amastigotes were also evaluated after treatment with free sugiol at the IC_50_ concentration. Treatment with free sugiol led to a remarkable presence of lysosomes, as well as an abnormal nucleus, in the macrophages compared to the untreated control. Disorganization in the ER was also observed in these macrophages (Figure 10D). Regarding the amastigotes, multiple nuclei, cytoplasmic and mitochondrial vacuolization, as well as a discrete mitochondrial swelling were observed after treatment with free sugiol (Figures 10E,F). YCWPs were readily identified in the macrophage cytosol, due to their size, and shape (Figures 10G–L). However, no signs of cytotoxicity on macrophages were observed after treatment with empty and sugiol-loaded YCWPs (Figures 10G–L). After treatment with YCWPs+sugiol, signs of amastigote degradation could be observed, as well as the presence of lysosomes (Figures 10J–L). It is worth noting that internalized YCWPs and amastigotes were located in the phagosomes and parasitophorous vacuoles, respectively (Figures 10H,I,L).

## Discussion

The activity of the diterpenoid sugiol was tested against *L. infantum* promastigotes over a time course of up to 72 h. Thus, the findings will be discussed sequentially for the time-points evaluated in order to explain the processes that occurred during treatment. It is important to state the similarity between the IC_50_ at 48 and 72 h, which was half the value compared to that at 24 h, which suggests that sugiol was more active during the log phase of parasite growth.

At 24 h of treatment, the induction of autophagy could be observed by MDC labeling. Autophagy is an early pro-survival response that occurs under stress conditions, which can be followed by cell death either by apoptosis or necrosis (Denton et al., 2015). Sengupta et al. (2011) found that the autophagic process in *Leishmania* treated with cryptolepine functioned as a survival mechanism and the tested drug caused cell death by inhibiting this process. Thus, the induction of autophagy as a pro-survival mechanism could explain the mild sugiol activity at 24 h of treatment.

The increase of intracellular calcium, a second messenger essential in several cellular processes, was observed in promastigotes as a consequence of sugiol treatment. In Trypanosomatid parasites, calcium can be released from the ER, mitochondria, and acidocalcisomes. TEM micrographs showed swollen mitochondria and ER dilation. Dilation of the ER lumen is a well-documented ultrastructural response to ER stress (Schönthal, 2012), which plays an important role in enhancing of ROS production and cytosolic Ca^2+^ concentration (Dolai et al., 2011). Changes in ROS mitochondrial production could be confirmed in the present study at 48 h of treatment.

High cytosolic Ca^2+^ levels induced by sugiol in this study might have stimulated mitochondria Ca^2+^ uptake resulting in mitochondrial membrane potential dysfunction (Mukherjee et al., 2002; Moreno and Docampo, 2003). The reduction of MDC fluorescence over time corroborated studies that show the prevention of autophagy due to the mitochondria Ca^2+^ uptake (Mallilankaraman et al., 2012; Cárdenas et al., 2016). This hypothesis explains the MDC fluorescence reduction at 72 h of treatment since the depletion of the intracellular calcium level was time-dependent.

According to Mehta and Shaha (2004), the hyperpolarization of the mitochondrial membrane potential after ROS production could be related to the apoptosis-like process occurring in *Leishmania donovani* through the inhibition of respiratory chain complex I. Complex I is the enzyme responsible for reducing ubiquinone with two electrons provided by NADH in the mitochondrial respiratory chain. It is common to observe an increase in ROS before the complex I inhibition, followed by the mitochondrial membrane hyperpolarization, which together may precipitate the development of apoptosis (Mehta and Shaha, 2004; Sousa et al., 2018). However, the loss of plasma membrane integrity and the absence of DNA fragmentation contradict the theory of an early apoptosis-like process developing in this case.

It should be acknowledged that the exposure of phosphatidylserine in *Leishmania* promastigotes as a marker of apoptosis-like cell death is still a controversial subject. Although Weingärtner et al. (2012) have shown absence of PS in these parasites, Imbert et al. (2012) demonstrated the presence of PS in the membrane of *Leishmania* promastigotes in addition to other phospholipids. However, researches agreed that there are apoptotic features in *Leishmania* spp., such as cell rounding, chromatin condensation, DNA fragmentation, and mitochondrial depolarization (Jiménez-Ruiz et al., 2010; Adak, 2015; Saini et al., 2017; Shadab et al., 2017). Our results showed that sugiol induced only one of these features – the cell rounding. However, Annexin-V-FITC and PI assay indicated the cell death by secondary necrosis, since some authors affirm that primary necrotic cells with intensely damaged membranes stain rapidly and strongly with PI and may not exhibit annexin V-FITC staining (Schutte et al., 1998; Wlodkowic et al., 2011; Martínez-Espinosa et al., 2015), which was not observed after sugiol treatment. Endorsing the secondary necrosis hypothesis, sugiol caused mitochondrial membrane potential hyperpolarization at 72 h (Vanden Berghe et al., 2010). Similar results associating mitochondrial membrane potential hyperpolarization, the increase in intracellular calcium level, ROS production, and necrotic cell death were described by Gómez-Pérez et al. (2014) in *Leishmania* spp. promastigotes after treatment with the bis-pyridinium derivative. In addition, as the *L. infantum* promastigotes did not show leaking of intracellular content or cell membrane damage, as assessed by EM, primary necrosis is unlikely.

All these findings suggest that the calcium-induced autophagy triggers sugiol antileishmanial activity at 24 h. After 48 h of incubation, promastigotes suffered structural and metabolic changes, such as an elevation in ROS levels and annexin V and PI staining, suggesting the development of a secondary necrosis process. Within 72 h of treatment, mitochondrial membrane hyperpolarization and substantial lipid peroxidation had developed. This chronology is in accordance with the time-dependent sugiol effect, leaving no doubt of its antileishmanial activity.

Previous studies have highlighted a potential issue with the delivery of sugiol due to its low water solubility (Bredenberg and Gripenberg, 1954; Sengupta et al., 1960). This would be a hinderance in the investigation of the antileishmanial activity *in vivo*. In order to study the action of sugiol against *L. infantum* amastigotes inside host cells a new delivery method was developed. In this work, sugiol was entrapped in glucan-rich particles obtained from *Saccharomyces cerevisiae* yeast cell walls (YCWPs) for efficient delivery into macrophages.

To entrap drugs within micro and nanoparticles is a strategy to improve the formulation-performance ratio to enhanced dissolution, and, consequently, augmenting treatment efficacy (Merisko-Liversidge and Liversidge, 2008). For this reason, after confirmation of sugiol antileishmanial potential, further tests of the effects against parasites and macrophages were carried out using this compound entrapped inside microparticles obtained from “baker's yeasts,” an affordable raw material. The method used in this study to extract the intracellular material from yeast was specific for the conservation of alkali-insoluble glucan or β-1,3-D-glucan of the cell wall (Fleet and Manners, 1976). YCWPs were able to retain water, according to the TEM micrographs. The porous nature of YCWPs ensured the encapsulation of sugiol molecules by simple diffusion, as expected (Paramera et al., 2014). But the dehydration promoted by the acetone during the extraction process could be noticed in SEM micrographs, leading to a reduction of the pore size, which may have limited the available space to entrap sugiol. Similar findings have also been described by Pan et al. (2015).

Drug solubility is also a critical point in carrier system development because it determines the drug loading in the encapsulated formulations and interferes in the drug bioavailability (Burton et al., 2002; Shaw and Carter, 2014). Bearing in mind future *in vivo* assays and the necessity to direct encapsulated systems toward the organs affected by visceral leishmaniasis, the use of glucan particles derived from yeast containing biologically active molecules would be an interesting treatment strategy. The targeting to the organs affected by visceral leishmaniasis would occur after oral administration, in which YCWPs are phagocytosed by M cells in intestinal Peyer's patches for posterior transfer to the macrophages associated with the lymphoid tissue in the intestine. These macrophages migrate to the spleen, lymph nodes, and bone marrow, which are the central tissues for *L. infantum* infection (Yan et al., 2005). Thus, YCWPs are able to carry the bioactive molecule and promote the immunological response in these sites.

For obligate intracellular microorganisms, such as *Leishmania* spp., the host immune response plays an essential role in disease dynamics. These responses are disrupted by the parasites in the infected cells, due to several mechanisms that ensure an ideal environment for nutrient availability and parasite protection. Studies have shown that β-glucan from different natural sources acts against *Leishmania* spp. due to the immunomodulatory stimulus (Ghosh et al., 2013; Shivahare et al., 2016). This corroborates the antileishmanial activity against intracellular amastigotes from empty YCWPs found in this study.

Besides the immunomodulatory effect, YCWPs were investigated in this study as drug carriers. To target *Leishmania* spp. as well as other intracellular parasites, an active molecule needs to cross at least three membranes: the host cell, the parasitophorous vacuole, and the amastigote (Basore et al., 2015). The natural transposition of the first barrier occurs through the recognition of glucan from YCWPs by the dectin-1 receptor on macrophages. This mechanism is responsible for the phagocytosis of YCWPs followed by an inflammatory response and consequent YCWPs degradation (Herre et al., 2004; Taylor et al., 2007).

YCWPs destruction is a natural macrophage competence since the macrophages are effectors cells of the innate immune response, with high capacity to release large quantities of highly reactive cytotoxic oxidants (Laskin et al., 2011). Therefore, only within the macrophages, YCWPs could be degraded and sugiol would be released. YCWPs and amastigotes could be observed in phagosomes and parasitophorous vacuoles in TEM micrographs, respectively. Previous studies showed that parasitophorous vacuoles could fuse with each other or with phagosomes containing macromolecules, colloids, inert particles, including yeast cell wall or zymosan (Alexander and Vickerman, 1975; Berman et al., 1981; Rabinovitch et al., 1985; Veras et al., 1996; Collins et al., 1997; Veras, 2004; Real et al., 2008, 2010). Moreover, *Leishmania* infection delays lysosome recruitment, hindering the maturation of the macrophage phagolysosome to ensure the intracellular parasite survival (Moradin and Descoteaux, 2012). Lysosome recruitment, whenever sugiol was present, may suggest a possible recovering of the natural macrophage defenses that were disabled by the *L. infantum* infection.

The antileishmanial activity and structural changes in amastigotes, as well as signs of YCWPs degradation in the infected macrophages treated with YCWPs+sugiol may suggest that sugiol was released from the YCWPs.

Despite the antileishmanial activity of YCWPs+sugiol against amastigotes, the absence of dectin-1 on the surface of promastigotes prevented the antileishmanial activity of empty and sugiol-loaded YCWPs against this extracellular form of the parasite and revealed that sugiol was not released in the aqueous medium. Thus, the phagocytosis of YCWPs by macrophages is essential for the release and activity of YCWPs+sugiol.

In summary, free sugiol was active against promastigotes and amastigotes of *L. infantum*. Evaluation of antileishmanial activity of sugiol over a time course revealed a sequence of the cellular events induced by sugiol that promoted cell death by secondary necrosis in promastigotes. Moreover, the utilization of YCWPs as a strategy to deliver water-insoluble drugs inside the infected cell was successfully applied in this study and the sugiol released inside the target cell was active against the *L. infantum* amastigotes. The encapsulation of other water-insoluble molecules using *S. cerevisiae* cell wall particles could be an ideal and low-cost alternative to direct bioactive molecules against intracellular parasites.

## Author Contributions

All authors contributed in the concept and design the study. DS planned the experiments, collected, analyzed, interpreted the data, and wrote the manuscript. HV, ES, TU-N, BD-F, MS, and CN supported the collect and the biological data analysis. NF contributed in the cell cycle methodology performing, data interpretation, supported the writing of the manuscript, and critically reviewed the text. ZD and ER-F planned, execute the obtaining and provided the sugiol molecule. AR and OB proposed, planned, and supported the data acquisition related to the yeast cell wall particles. CN, MS, and OB coordinated all stages of the work, concepted, designed, and drafted the study, interpreted the results, reviewed critically, and corrected of the manuscript.

### Conflict of Interest Statement

The authors declare that the research was conducted in the absence of any commercial or financial relationships that could be construed as a potential conflict of interest.
