# Supplementary figures and images for: Activity and Cell-Death Pathway in Leishmania infantum Induced by Sugiol: Vectorization Using Yeast Cell Wall Particles Obtained From Saccharomyces cerevisiae
Source: Front Cell Infect Microbiol. 2019 Jun 14;9:208. doi: 10.3389/fcimb.2019.00208 (PMC6587907; doi:10.3389/fcimb.2019.00208)

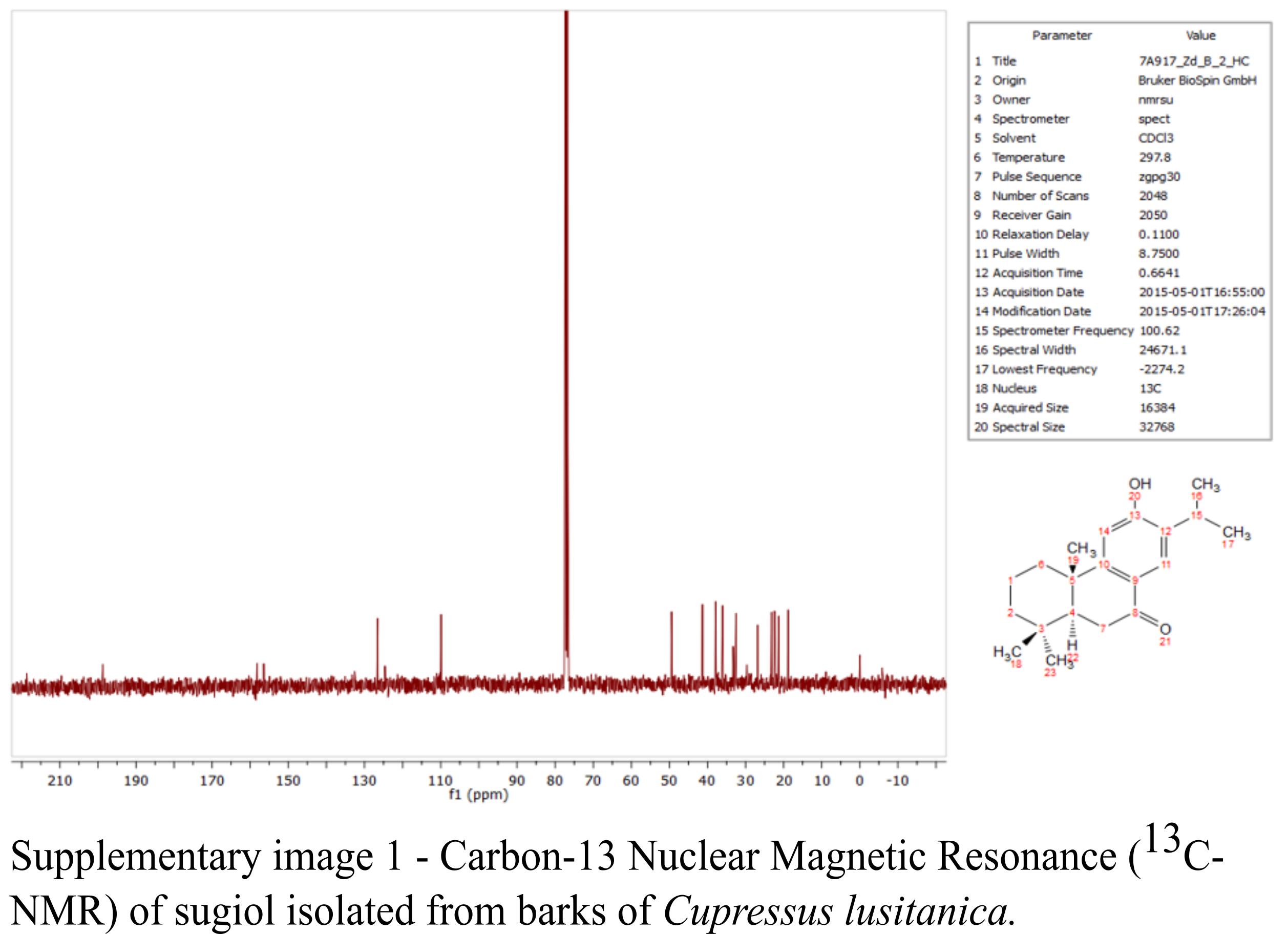

Supplement: Supplementary file 1 [file Image_1.TIF]

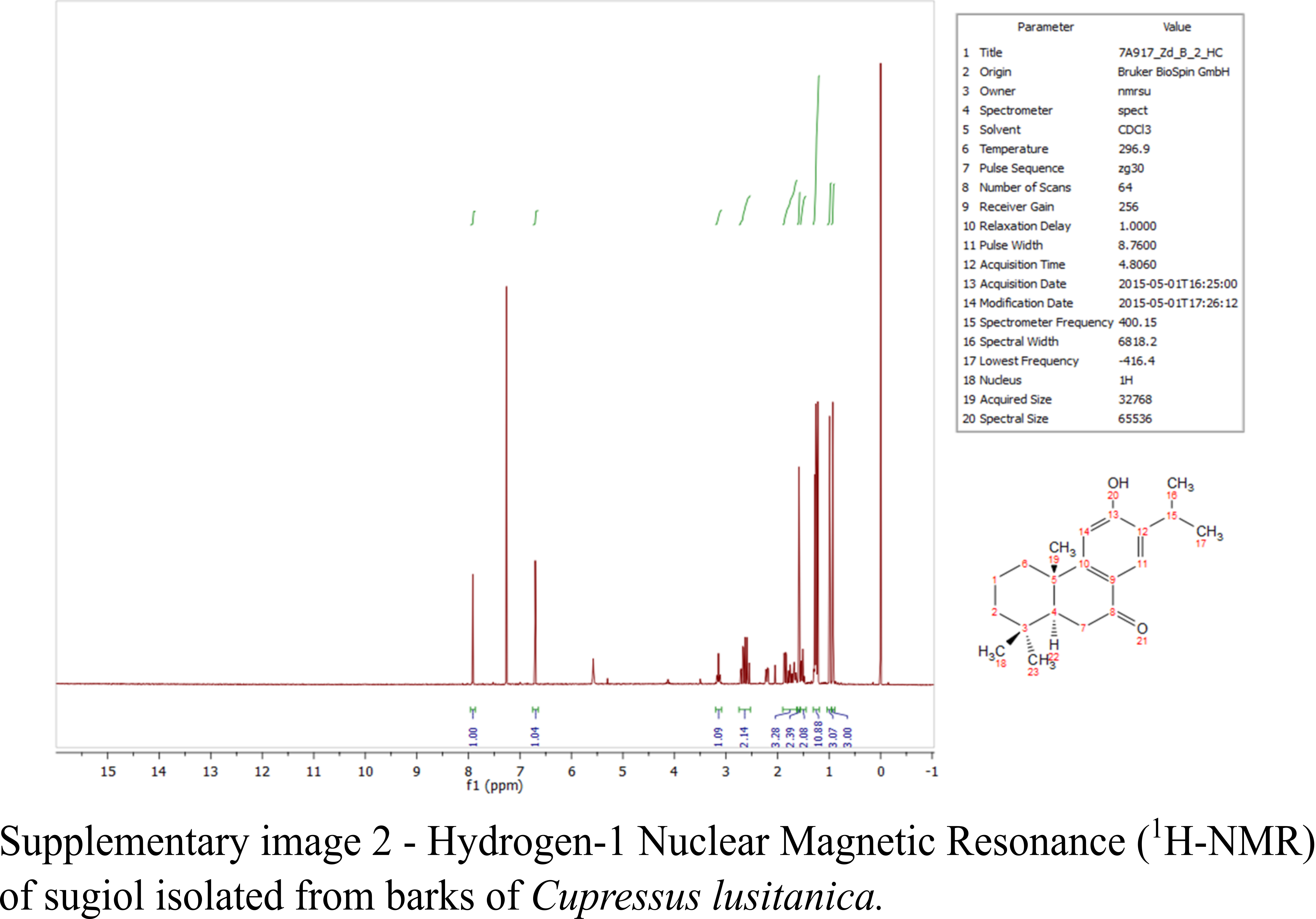

Supplement: Supplementary file 2 [file Image_2.TIF]
